# *MECP2* Isoform-Specific Vectors with Regulated Expression for Rett Syndrome Gene Therapy

**DOI:** 10.1371/journal.pone.0006810

**Published:** 2009-08-27

**Authors:** Mojgan Rastegar, Akitsu Hotta, Peter Pasceri, Maisam Makarem, Aaron Y. L. Cheung, Shauna Elliott, Katya J. Park, Megumi Adachi, Frederick S. Jones, Ian D. Clarke, Peter Dirks, James Ellis

**Affiliations:** 1 Developmental and Stem Cell Biology Program, SickKids Hospital, Toronto, Ontario, Canada; 2 Department of Molecular Genetics, University of Toronto, Toronto, Ontario, Canada; 3 Experimental Neurobiology, The Neurosciences Institute, San Diego, California, United States of America; 4 Institute of Medical Science, University of Toronto, Toronto, Ontario, Canada; Universidade Federal do Rio de Janeiro (UFRJ), Instituto de Biofísica da UFRJ, Brazil

## Abstract

**Background:**

Rett Syndrome (RTT) is an Autism Spectrum Disorder and the leading cause of mental retardation in females. RTT is caused by mutations in the Methyl CpG-Binding Protein-2 (*MECP2*) gene and has no treatment. Our objective is to develop viral vectors for *MECP2* gene transfer into Neural Stem Cells (NSC) and neurons suitable for gene therapy of Rett Syndrome.

**Methodology/Principal Findings:**

We generated self-inactivating (SIN) retroviral vectors with the ubiquitous EF1α promoter avoiding known silencer elements to escape stem-cell-specific viral silencing. High efficiency NSC infection resulted in long-term EGFP expression in transduced NSC and after differentiation into neurons. Infection with Myc-tagged *MECP2*-isoform-specific (E1 and E2) vectors directed MeCP2 to heterochromatin of transduced NSC and neurons. In contrast, vectors with an internal mouse *Mecp2* promoter (MeP) directed restricted expression only in neurons and glia and not NSC, recapitulating the endogenous expression pattern required to avoid detrimental consequences of *MECP2* ectopic expression. In differentiated NSC from adult heterozygous *Mecp2^tm1.1Bird^*+/− female mice, 48% of neurons expressed endogenous MeCP2 due to random inactivation of the X-linked *Mecp2* gene. Retroviral *MECP2* transduction with EF1α and MeP vectors rescued expression in 95–100% of neurons resulting in increased dendrite branching function *in vitro*. Insulated *MECP2* isoform-specific lentiviral vectors show long-term expression in NSC and their differentiated neuronal progeny, and directly infect dissociated murine cortical neurons with high efficiency.

**Conclusions/Significance:**

MeP vectors recapitulate the endogenous expression pattern of MeCP2 in neurons and glia. They have utility to study MeCP2 isoform-specific functions *in vitro*, and are effective gene therapy vectors for rescuing dendritic maturation of neurons in an *ex vivo* model of RTT.

## Introduction

Rett Syndrome (RTT) is an X-linked progressive neurological disorder affecting 1 in every 10,000 female births that leads to severe mental retardation. RTT patients develop normally up to 6–18 months of age, when they start to develop symptoms including loss of speech and purposeful hand movements, seizures, respiratory abnormalities, anxiety and autism [1]. RTT is caused by mutations in the methyl-CpG binding protein-2 (*MECP2*) gene. MeCP2 has two NLS (Nuclear Localization Signals) and three principal domains; the Methyl DNA Binding Domain (MBD), the Transcriptional Repression Domain (TRD) and a C-terminal domain. Implicated as both an activator and a repressor [2], [3], MeCP2 binds via its MBD to methylated CpG dinucleotides adjacent to A/T sequences [4] and recruits HDAC1/2 (Histone Deacetylase 1 and 2) and transcriptional regulator Sin3A [5], [6]. The TRD can also function as a nonspecific DNA binding domain [7]. To fully assert its gene repression activity on target genes, MeCP2 interacts with the Brahma component of SWI/SNF chromatin remodeling complex [8], HP1 isoforms (Cbx1, 3, 5) [9], and DNMT1 (DNA methyltransferase 1) [10]. In addition, interactions with RNA-binding protein YB1 (Y box-binding protein 1) [11] forms complexes that modulate RNA splicing patterns. *MECP2* mutations have different impacts in protein function depending on where the mutation lies. For example, distinct mutations within the MBD result in structural protein changes that alter protein folding and DNA interaction abilities of MeCP2 [7], [12]. Although the function of MeCP2 through these interactions is not clearly established, they highlight its multiple roles in gene repression, chromatin condensation/remodeling and RNA splicing.

MeCP2 isoforms E1 and E2 are generated by alternative splicing of exon 2 to produce proteins with differing N termini [13]. *MECP2* transcripts are expressed almost ubiquitously with higher expression of the E1 isoform in the brain [14], but no MeCP2 expression is detected in Neural Stem Cells (NSC) grown as neurospheres. Although MeCP2 is widely expressed, RTT symptoms are primarily neuronal and confirmed MeCP2 targets in neurons include BDNF (Brain Derived Neurotrophic Factor) [15]–[17] and DLX5 (Distal-less homeobox 5) [18]. It was recently reported that MeCP2 is expressed at low levels in glia. In particular GFAP+ astrocytes support normal neuronal growth, but MeCP2-deficient astrocytes have a non-cell autonomous effect on dendritic morphology of cocultured neurons exerted through aberrant secretion of a soluble factor [19]. Thus it is important to maintain MeCP2 expression in both neurons and glia.

Currently, there is no effective treatment for Rett Syndrome. However, it has been shown that reactivation of the *Mecp2* gene after the onset of disease in RTT mouse models rescues the phenotype [20], [21]. This finding raises gene therapy prospects by delivering *MECP2* to the affected neurons and glia or their progenitors. Retroviral and lentiviral vectors integrate into the genome and provide stable gene transfer. However, these vectors are often subject to transcriptional silencing in stem cells, and when silent are bound by MeCP2 [22]–[26] and other repressor complexes. Thus these vectors may also be silenced in NSC, consequently limiting their gene transfer application for gene therapy of neurological diseases. Therefore, it is important to study vector expression in stem cell systems tailored for gene transfer. Delivery of the *MECP2* gene by direct viral infection, or by transplantation of engineered NSC into specific regions of the brain to migrate and differentiate into neurons and glia, may ameliorate Rett Syndrome symptoms. However, the vectors must be designed to direct long-term expression in the correct cell types.

We have designed *MECP2* isoform-specific retroviral vectors with ubiquitous (EF1α) or endogenous *Mecp2* (MeP) internal promoters. We demonstrate long-term expression of the EF1α vectors after transduction of embryonic and adult murine NSC and differentiation into neurons. The MeP vector recapitulates endogenous MeCP2 expression in neurons and glia but not NSC, and thus is well suited for gene therapy. Retroviral gene transfer of the E1 isoform into *Mecp2^tm1.1Bird^*+/− NSC directed expression in 95% of the differentiated cells, and demonstrates a functional role for MeCP2-E1 in regulating dendrite length and branching during morphological maturation of neurons *in vitro*. Equivalent lentiviral EF1α vectors express long-term in NSC and their progeny neurons, while lentiviral MeP vectors express *MECP2* isoforms after direct gene transfer into cortical neurons and glia. These *MECP2* vectors will facilitate functional studies on the isoforms and ultimately have applications for RTT gene therapy.

## Results

### Efficient transduction and long-term expression of MECP2 retroviral vectors in NSC

**Figure 1:**
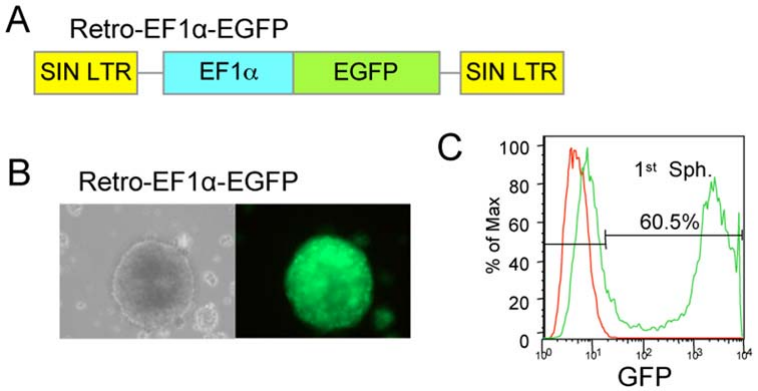
Efficient transduction of NSCs by a retroviral vector expressing EGFP. A) Schematic of the retroviral vector expressing EGFP under the control of the EF1α promoter. SIN LTR: Self-inactivating Long Terminal Repeat. B) Primary neurospheres infected with Retro-EF1α-EGFP expressed EGFP under fluorescence microscope. C) Flow cytometry analysis of infected primary neurospheres shows 60% EGFP positive cells.

To create retroviral vectors for gene delivery into NSC, we combined the self-inactivating (SIN) HSC1 retroviral backbone with a strong ubiquitous 1.3 kb EF1α promoter [27] suitable for high expression in stem cells. SIN vectors carry a deleted LTR (Long Terminal Repeat) promoter that results in transcriptional initiation exclusively from the internal promoter. To assess expression of control Retro-EF1α-EGFP vector (Figure 1A), isolated NSC from embryonic (E14) mouse forebrain were grown in the presence of rhEGF and bFGF, and dissociated cells were infected. EGFP expression from transduced NSC was detectable within 48 h of infection by live imaging (Figure 1B) and persisted during neurosphere formation in 60% of cells as detected by flow cytometry (Figure 1C).

**Figure 2:**
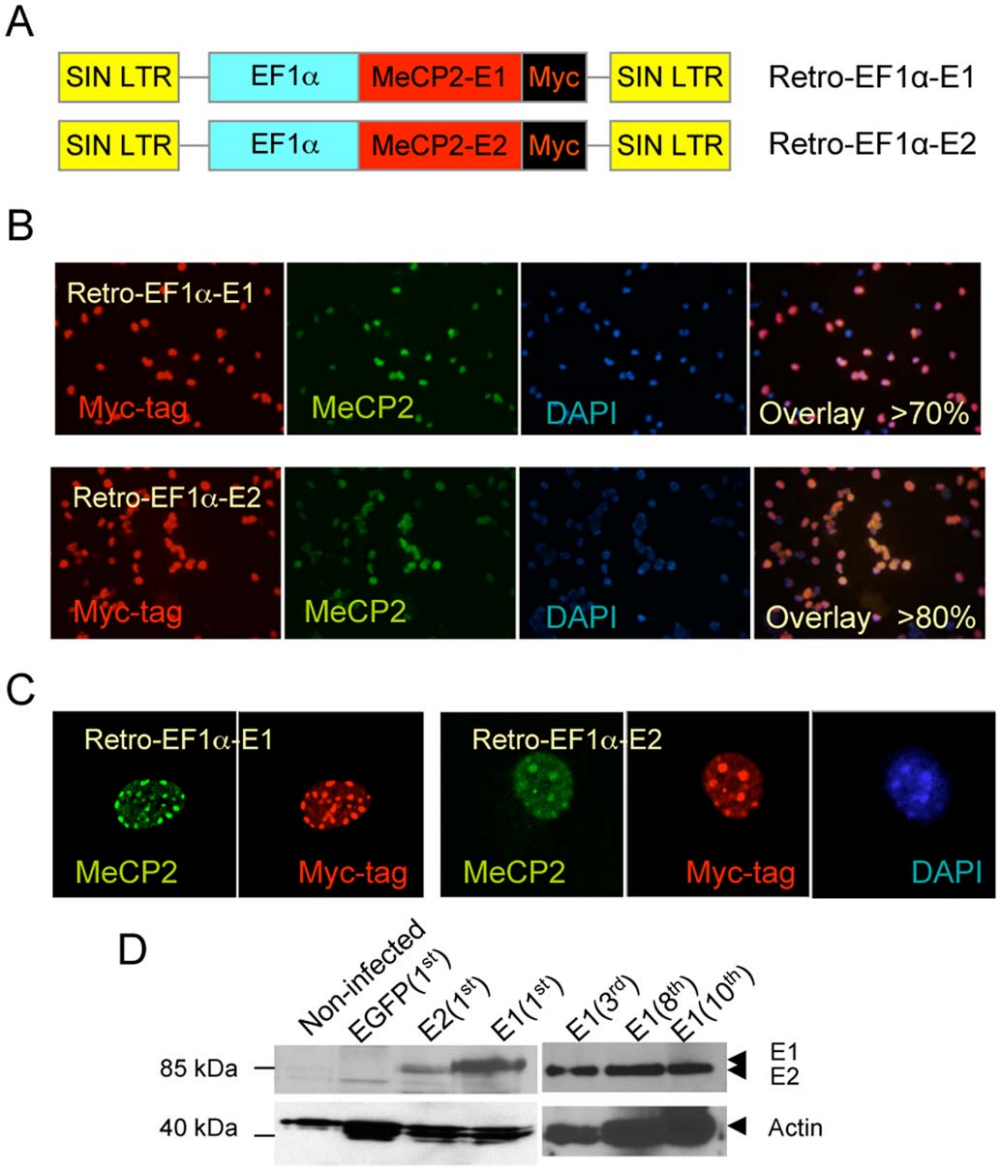
Efficient transduction of NSCs by retroviral vectors expressing MeCP2 isoforms. A) Schematic of the retroviral vectors expressing human MeCP2 isoforms (E1 or E2) with Myc-tag under the control of the EF1α promoter. B) Ratio of Myc-tagged MeCP2 positive cells in dissociated embryonic NSCs after retroviral infection. C) Confocal microscope images of the infected NSCs show punctate staining with colocalization of Myc-tag and MeCP2 protein at DAPI-rich regions. D) Both E1 and E2 isoforms were detected in transduced NSCs (1^st^ sphere for E2 isoform, and 1^st^, 3^rd^, 8^th^, and 10^th^ spheres for E1 isoform) by Western blot (WB) using anti Myc-tag antibody.

To avoid stem cell-specific silencing [27], [28], we did not include EGFP in the *MECP2* vectors. EGFP cDNA contains 60 CpG dinucleotides within the coding sequence and could be subject to silencing via DNA methylation that could become a target for MeCP2 [27]. We therefore generated Retro-EF1α-*MECP2*-E1 or -E2 vectors using Myc-tagged cDNAs to distinguish them from the endogenous protein (Figure 2A). Immunofluorescence (IF) staining of dissociated NSC with anti Myc-tag and anti MeCP2 antibodies showed that >70% to >80% of cells are infected with Retro-EF1α-E1 or Retro-EF1α-E2 respectively (Figure 2B). Confocal single cell images showed colocalization of Myc-tag and MeCP2 signals at DAPI-rich heterochromatic regions of the nucleus (Figure 2C). The specificity of MeCP2-myc staining is clear in adjacent infected and noninfected cells (data not shown). The molecular weight of MeCP2 in Western blots (WB) is between 70 kDa and 100 kDa, depending on the cell type, antibody used and post-translational modifications. We detected both MeCP2 isoforms with the Myc-tag at 80–85 kDa in infected primary transduced neurospheres by WB (Figure 2D). We conclude that *MECP2* isoform-specific retroviral vectors transduce embryonic NSC with high efficiency directing MeCP2 correctly to heterochromatic regions of the nucleus.

To examine long-term vector expression of the transduced genes, we monitored EGFP expression over 10 neurosphere passages by flow cytometry and did not detect any decrease in the percentage of EGFP expressing cells. We also collected protein extracts from Retro-EF1α-E1 infected NSC at the 3^rd^, 8^th^ and 10^th^ neurosphere passages and by WB detected MeCP2-myc expression at all passages (Figure 2D). These results indicate that infected NSC express transduced genes in long-term cultures, and high levels of expression are obtained from the EF1α promoter. Moreover, Retro-EF1α-EGFP and E1 *in vitro* transduced NSC injected into WT brain tissue in culture resulted in EGFP expressing cells within the brain slices (Figure S1) demonstrating transduced gene delivery via NSC injection into the brain microenvironment.

### Mecp2 promoter recapitulates endogenous MeCP2 expression in neurons and in glia

**Figure 3:**
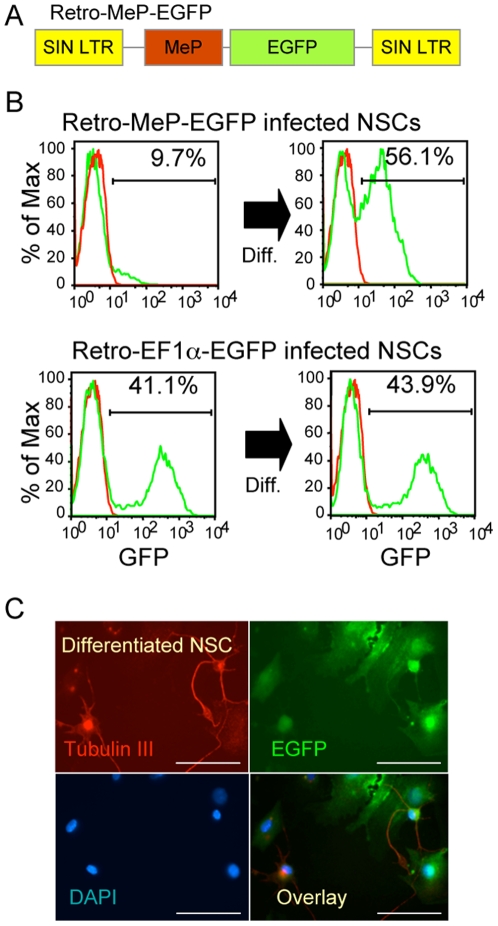
Regulated MeP promoter expresses EGFP in neurons but not NSCs. A) Schematic of the retroviral vector expressing EGFP under the control of MeCP2 (MeP) promoter. B) Flow cytometry analysis shows that MeP promoter only expressed EGFP after 7 day differentiation of NSCs into neurons, whereas control Retro-EF1α-EGFP vector expressed in both undifferentiated and differentiated NSCs. C) Immunofluorescence staining images show that Tubulin III positive neurons express EGFP after differentiation of NSCs infected with Retro-MeP-EGFP. Scale bars represent 50 µm.

MeCP2 overexpression in mice causes severe motor dysfunction when expressed in a WT background, while neuronal-specific transgene expression in *Mecp2* mutant mice rescues the RTT phenotype [29]. It is therefore critical to maintain restricted levels of MeCP2 by employing endogenous regulatory elements. A previously reported *Mecp2* promoter [30], that we refer to as MeP, was used to generate EGFP (control) and *MECP2*-E1 or -E2 retroviral vectors for regulated expression in neuronal tissue. Retro-MeP-EGFP (Figure 3A) vector did not express EGFP in transduced NSC as expected (Figure 3B, left). For potential RTT gene therapy, MeCP2 must be expressed in affected neurons and their supporting glial cells. We therefore differentiated transduced NSC for 7 days in the presence of serum and withdrawal of rhEGF and bFGF. EGFP expression from Retro-MeP-EGFP vector was induced in the resulting cells (56%) as detected by flow cytometry (Figure 3B, right) and IF (Figure 3C). WB of protein extracts from transduced NSC confirmed that both isoforms express long-term from the EF1α promoter, but expression from the MeP promoter is negligible until NSC are induced to differentiate (data not shown). To confirm the neuronal source of MeP directed EGFP in differentiated NSC, we performed IF and found EGFP-expressing neuronal (Figure 3C) and non-neuronal cells based on Tubulin III ( = βIII Tubulin, or also known as Tubb3) staining.

**Figure 4:**
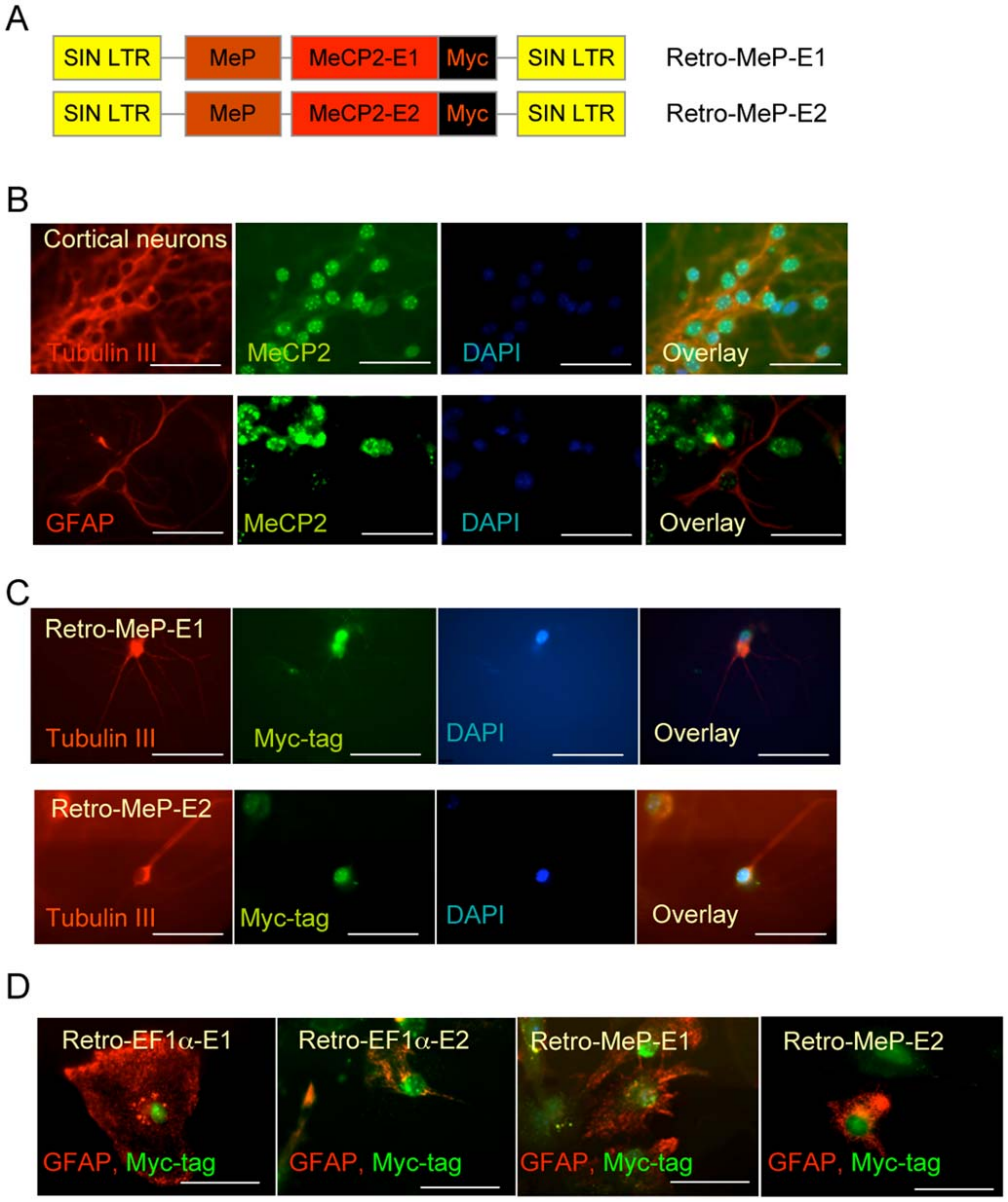
MeP promoter is active in neurons and glia. A) Schematic of the retroviral vectors expressing human MeCP2 isoforms (E1 or E2) with Myc-tag under the control of MeCP2 (MeP) promoter. B) Expression of endogenous MeCP2 protein in cortical neurons and glia isolated from E18 mouse brain. C) MeP promoter expressed MeCP2-E1 (top) or E2 (bottom) protein in Tubulin III positive neurons after 7 days differentiation of infected NSCs. D) EF1α promoter and MeP promoter expressed MeCP2-E1 or E2 protein in GFAP positive glia after 7 days differentiation of infected NSCs. Scale bars represent 50 µm.

MeCP2 has been reported to be expressed in neurons and to a lower level in glia [19], [31], [32], and the *Mecp2* promoter is known to be primarily neuronal in transgenic mice [30], [33]. Based on the Retro-MeP-EGFP expression pattern, we hypothesized that Retro-MeP-*MECP2*-E1 and -E2 vectors (Figure 4A) may in fact recapitulate low-level endogenous MeCP2 expression in non-neuronal cells. We first investigated endogenous MeCP2 expression after *in vitro* differentiation of embryonic NSC. MeCP2 was detectable at D7 but not at D0 in Tubulin III+ neurons and in most differentiated cells (not shown). Further IF for GFAP, which is expressed in astrocytes, revealed that MeCP2 is expressed in the nuclei of glia differentiated from both embryonic and adult NSC (not shown). To address whether *in vivo* differentiated GFAP+ glia express MeCP2, we dissociated neurons and glia from the forebrain of E18 mouse embryos. While neurons strongly expressed nuclear MeCP2 colocalized with DAPI signals (Figure 4B), lower expression in glia was also detected (Figure 4B). We conclude that endogenous MeCP2 is expressed in neurons and to lower levels in GFAP+ glia as reported [19]. As expected, MeCP2-myc expression directed from the MeP promoter vectors was also detected in differentiated Tubulin III+ neurons derived from transduced NSC (Figure 4C). We confirmed expression in GFAP+ cells of transduced MeCP2 directed from both EF1α and MeP promoters, demonstrating that the MeP promoter is active in these cells (Figure 4D). Together, these data clearly show MeCP2 expression in GFAP+ cells derived *in vitro* and *in vivo* and that the MeP promoter recapitulates this restricted expression pattern.

### MECP2 retrovirus vector delivery into NSC of adult Mecp2^ tm1.1Bird^+/− female mice

**Figure 5:**
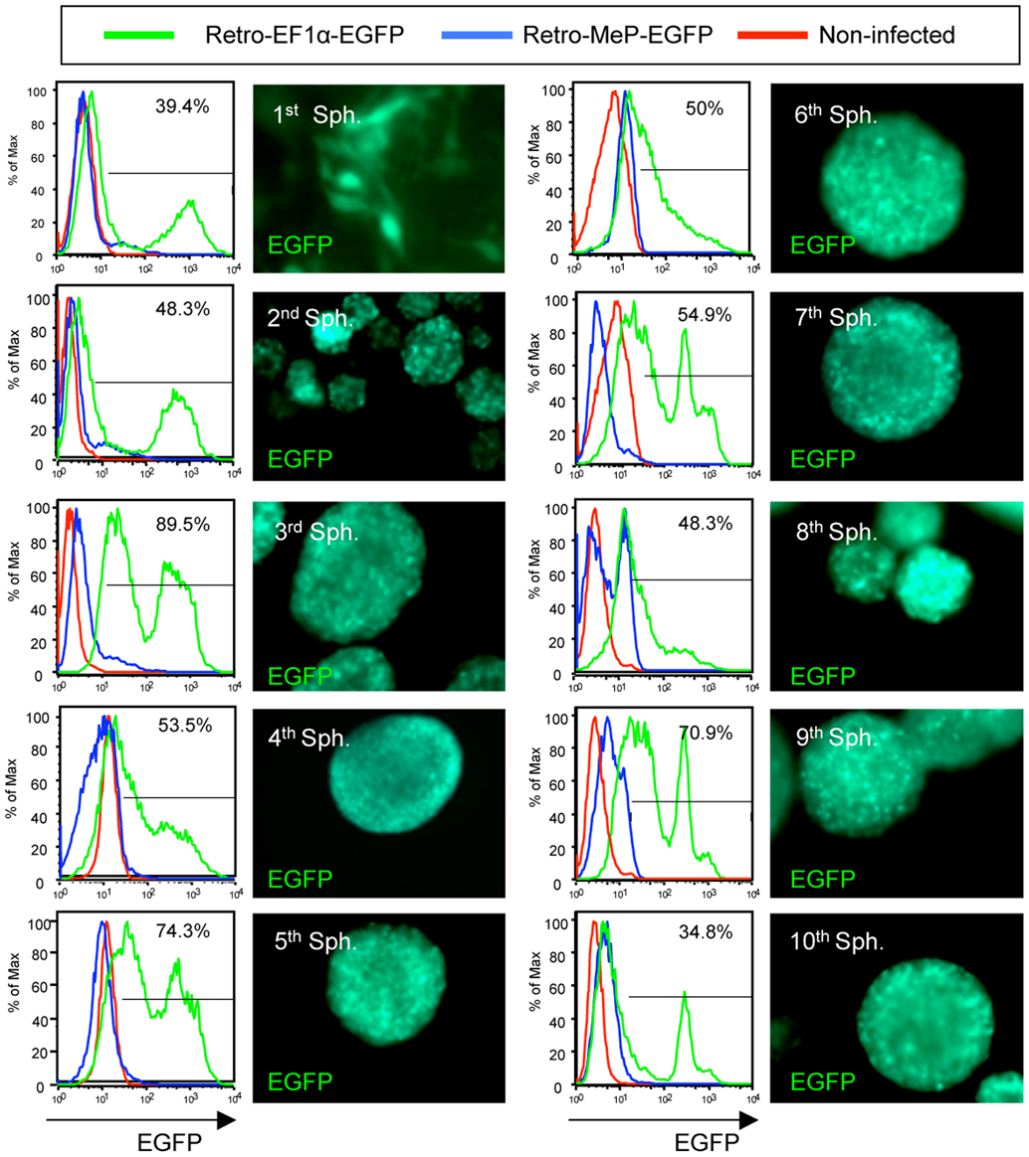
Long-term EGFP expression in NSCs from *Mecp2^tm1.1Bird^*+/− female mice. NSCs from adult *Mecp2^tm1.1Bird^*+/− female mice were infected with Retro-EF1α-EGFP (green), Retro-MeP-EGFP (blue), or non-infected control (red) and maintained in culture for 10 neurosphere passages (1 passage per week). Flow cytometry analysis (left) shows maintained expression from Retro-EF1-EGFP (green line) but no significant expression from Retro-MeP-EGFP (blue line). Live cell images (right) show EGFP expression in NSCs infected with Retro-EF1α-EGFP at indicated passages.

**Figure 6:**
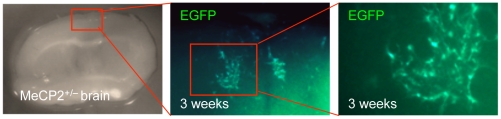
Long-term EGFP expression in *ex vivo* brain from *Mecp2^tm1.1Bird^*+/− female mice. NSCs were isolated from *Mecp2^tm1.1Bird^*+/− female brain and infected with Retro-EF1α-EGFP retroviral vector. EGFP expressing NSCs were injected into *ex vivo* slice cultures of *Mecp2^tm1.1Bird^*+/− female brain, and the EGFP positive NSCs that migrated were detected up to three weeks after NSC injection.

As RTT is a postnatal neurological disorder, it is important to confirm that retrovirus expression is maintained long-term in transduced adult NSC. To study retrovirus vector expression in an RTT model, one year old *Mecp2^tm1.1Bird^*+/− female mice showing RTT symptoms (Figure S2A) were used to isolate NSC. The cells were cultured for 20 neurosphere passages with no difficulty in generating new neurospheres (Figure S2B). Transduction of these cells with Retro-EF1α-EGFP resulted in EGFP expressing neurospheres that continued to express EGFP for 10 passages (Figure 5, green line) with comparable expression to primary spheres infected at the 10^th^ passage (39.4% and 34.8% respectively, Figure 5, upper right). Differentiation of these transduced NSC did not result in EGFP silencing (data not shown). As expected, no significant EGFP expression was detected in NSC transduced with Retro-MeP-EGFP (Figure 5, blue line). Injection of Retro EF1α-EGFP expressing transduced NSC into +/− brain slices in culture resulted in EGFP expressing cells that migrated and were detectable by live imaging monitored up to 3 weeks (Figure 6). Thus, RTT model NSC can be effectively transduced with retroviral vectors that maintain reporter gene expression.

**Figure 7:**
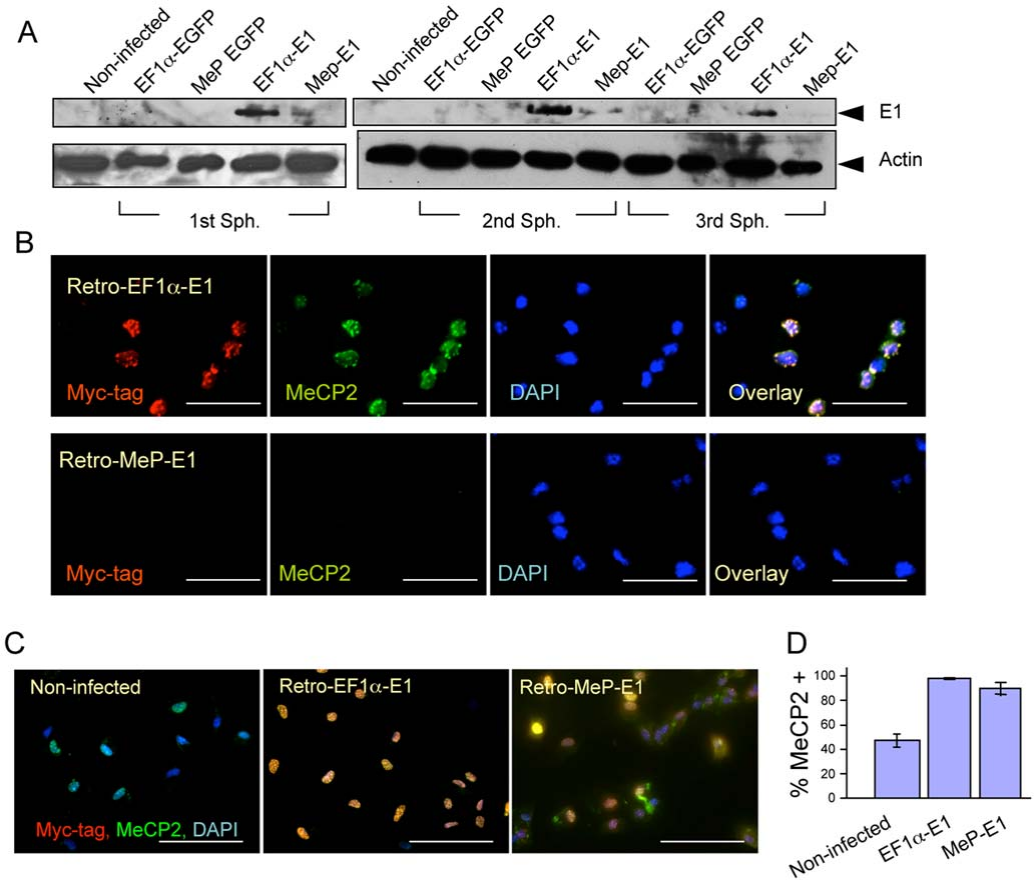
Rescue of MeCP2 expression in differentiated adult NSCs from *Mecp2^tm1.1Bird^*+/− mice. A) MeCP2-E1 protein from EF1α promoter was detected in whole-cell lysates of NSCs from *Mecp2^tm1.1Bird^*+/− female mice by WB using anti Myc-tag antibody. B) Undifferentiated NSCs from *Mecp2^tm1.1Bird^*+/− female mice express Myc-tagged MeCP2-E1 from EF1α promoter, but not from MeP promoter. C) Differentiated NSCs (for 14 days) from *Mecp2^tm1.1Bird^*+/− female mice show mosaic expression of endogenous MeCP2 (Non-infected). Myc-tagged MeCP2-E1 expression was detected from both EF1α promoter (Retro-EF1α-E1) and MeP promoter (Retro-MeP-E1). D) Percentage of MeCP2 positive cells shows approximately 50% of the cells expressing endogenous MeCP2 (Non-infected), whereas expression of MeCP2 protein in NSCs transduced by either Retro-EF1α-E1 or Retro-MeP-E1 shows 90–100%. Error bars represent SEM (standard error of the mean). Scale bars represent 50 µm.

To examine retrovirus vector expression of *MECP2*, the isolated +/− NSC were transduced with Retro-EF1α-E1 and Retro-MeP-E1. Robust expression was detected by WB from the ubiquitous EF1α promoter and negligible expression from the MeP promoter in the 1^st^, 2^nd^ and 3^rd^ passage (Figure 7A). Dissociated NSC transduced with Retro-EF1α-E1 but not with Retro-MeP-E1 showed punctate Myc-tag and MeCP2 signals (Figure 7B) confirming the WB data. To assess endogenous MeCP2 expression in +/− neurons, NSC were differentiated for 2 weeks. Since *MECP2* is an X-linked gene and undergoes X chromosome inactivation in females, 50% of the +/− neurons should express MeCP2. Both Tubulin III+ neurons and GFAP+ glia expressing MeCP2 were detected from the 1^st^ sphere (64.4%±4.7 MeCP2+ cells) and 5^th^ sphere (48.2%±5 MeCP2+ cells). Thus, the percentage of MeCP2 expressing cells was initially higher in primary spheres but adjusted to the expected 50% level after 5 passages. We speculate that MeCP2+ differentiated cells have a growth advantage *in vivo* that is evident in the primary neurospheres but is lost upon extended *in vitro* neurosphere culture.

We next tested the differentiation ability of transduced NSC with *MECP2-E1* constructs. Differentiation of these cells for two weeks resulted in Tubulin III+ neurons and GFAP+ glia showing nuclear Myc-tag staining colocalized with MeCP2 (data not shown). The signals were detectable from both the EF1α ubiquitous and MeP regulated promoters (Figure 7C). Quantification of MeCP2 or MeCP2-myc positive cells revealed that MeCP2-E1 expression reaches 95–100% of differentiated *Mecp2^tm1.1Bird^*+/− NSC (Figure 7D). These findings show that virtually all neurons derived from *Mecp2^tm1.1Bird^*+/− NSC express transduced *MECP2*. Such efficient NSC gene transfer and restricted expression pattern would be attractive for gene therapy of RTT.

### Transduced MeCP2-E1 promotes neuronal dendrite branching

**Figure 8:**
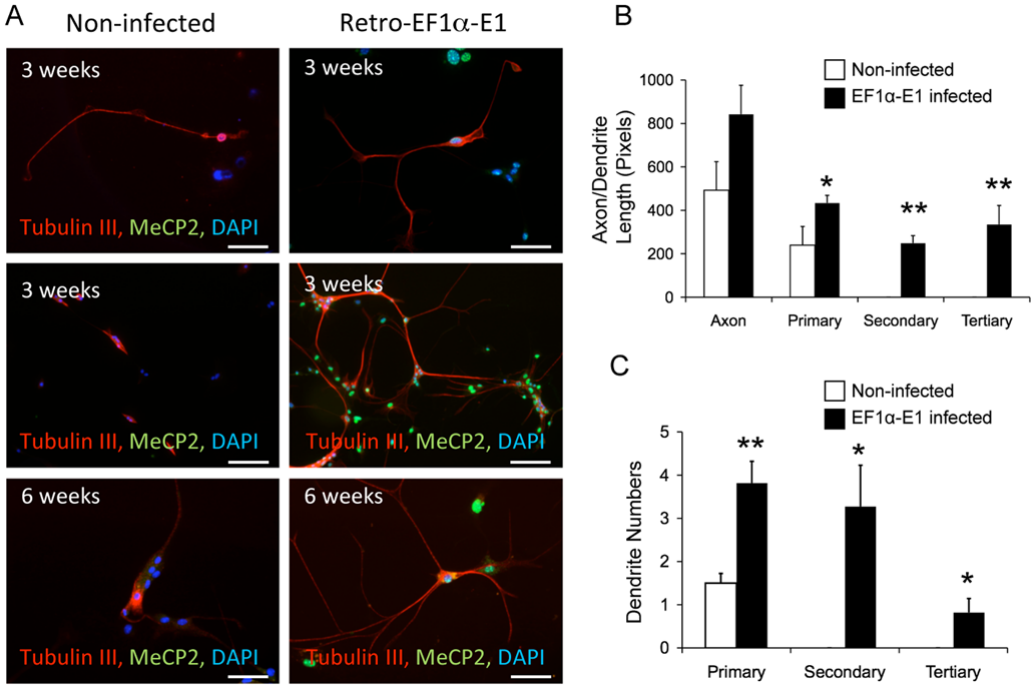
MeCP2 overexpression promotes dendrite length and branching. A) NSCs from *Mecp2^tm1.1Bird^*+/− mice (15^th^ passage for non-infected cells and 14^th^ passage for Retro-EF1α-E1 infected cells) were differentiated for 3 (top and middle panels) or 6 weeks (bottom panel), and stained for MeCP2 (green) and neuronal marker Tubulin III (red) for dendrite maturation analysis. Scale bars represent 50 µm. B–C) Quantification of axon and dendrite length (B) and dendrite numbers (C) shows that overexpression of MeCP2-E1 promoted dendrite formations. Error bars represent SEM. * indicate *P*<0.05, ** indicate *P*<0.005.

In neurons, MeCP2 is known to regulate glutamatergic synapse formation [34], neuronal maturation and dendrite arborization [32]. In order to test whether MeCP2-E1 overexpression directed from our retroviral vectors affects dendrite formation of differentiated neurons, we differentiated transduced NSC from the *Mecp2^tm1.1Bird^*+/− mice. Although two weeks of differentiation resulted in Tubulin III+ neurons, a longer differentiation period was required to detect dendrite branching. NSC differentiated for three weeks and six weeks were stained for the Tubulin III neuronal marker and MeCP2. By three weeks, differentiated neurons with primary and occasionally secondary dendrites were observed in the Retro-EF1α-E1 infected cells. In contrast, control non-infected neurons did not show any secondary dendrites and the length of their primary dendrites appeared smaller (Figure 8A, top). At this time, neuronal networks began to form in cells infected with the Retro-EF1α-E1 but not in non-infected cells (Figure 8A, middle). Further differentiation to six weeks promoted an increase in primary, secondary and tertiary dendrite numbers in the infected cells, while the non-infected cells did not develop secondary or tertiary dendrites (Figure 8A, bottom).

To quantify neuronal maturation after Retro-EF1α-E1 infection of +/− NSC, images of distinct well-separated neurons were analyzed using NeuronJ software. These measurements indicate that non-infected axon length is not significantly smaller than infected cells, but significant differences were observed for increased primary, secondary and tertiary dendrite lengths in the infected cells (Figure 8B). In terms of dendrite numbers, we also detected significant differences in general branching and formation of dendrites (Figure 8C). In general, our data support previous reports that MeCP2 is involved in neuronal maturation and dendrite formation. We conclude that the transduced MeCP2 has functional activity on the morphological maturation of neurons *in vitro*.

### Lentiviral vector delivery and long-term expression in neurons

Transduced NSC could migrate after delivery into the brain and produce neurons expressing MeCP2, but an attractive alternative strategy for RTT gene therapy via *MECP2* gene transfer is to develop lentiviral vectors that infect pre-existing neurons. We generated SIN lentiviral vectors containing the 500 bp dimer cHS4 core insulator in the LTRs [35]. *MECP2*-E1 or -E2 isoforms and an EGFP control were subcloned under the control of either the EF1α or MeP promoters (Figure S3A). These vectors were tested by infection of embryonic NSC, resulting in long-term expression of MeCP2 only from the EF1α promoter detected as punctate nuclear staining similar to that described with the *MECP2* retroviral vectors by IF (Figure S3B) and by WB (Figure S4). Upon differentiation of infected NSC, MeCP2-myc-positive neurons and glia were observed from both the EF1α and MeP promoters (Figure S5). The data presented here show that our lentiviral constructs express MeCP2 long-term in transduced neurospheres and after neuronal differentiation.

**Figure 9:**
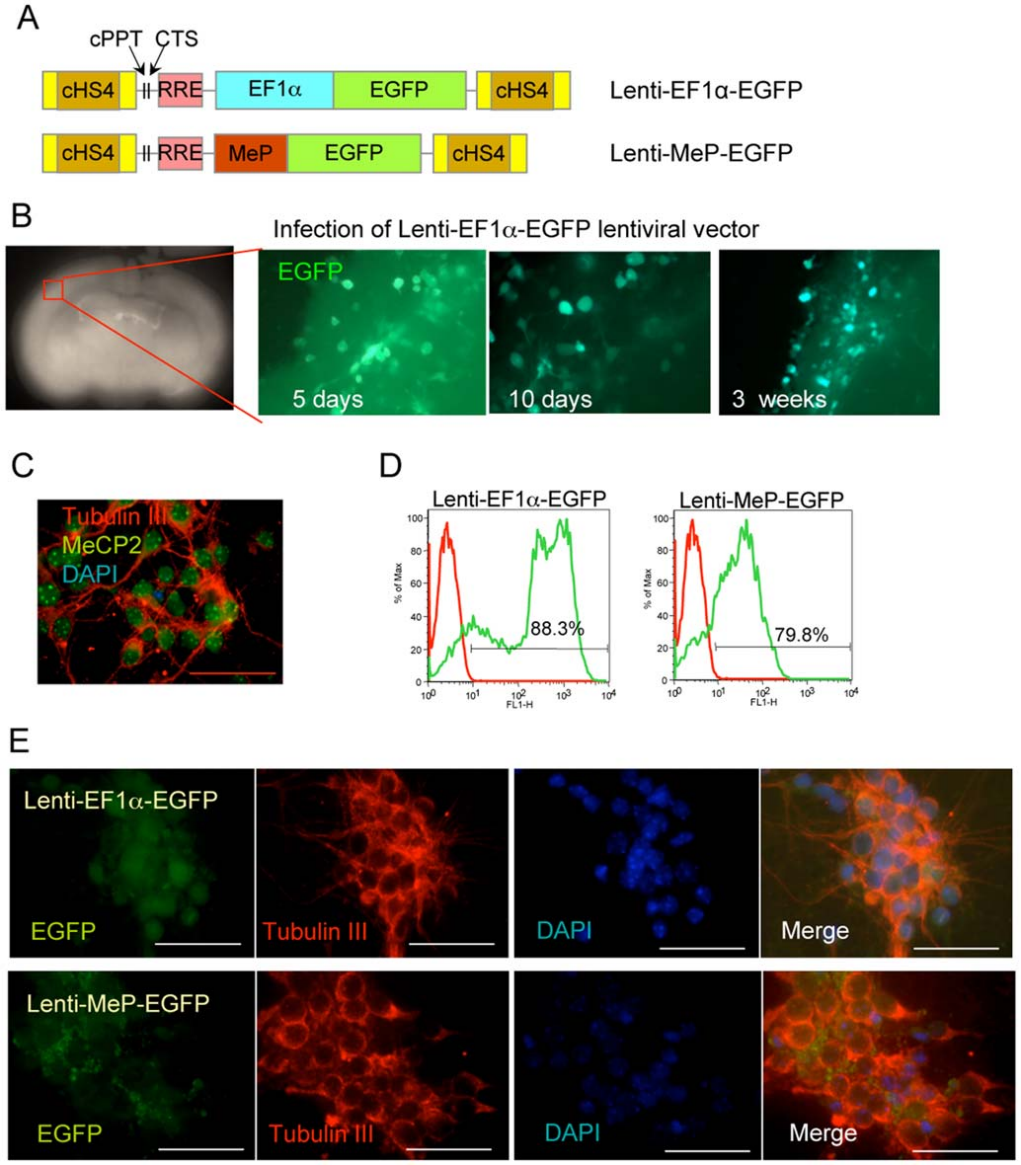
Efficient transduction of cortical neurons by lentiviral vectors expressing EGFP. A) Schematic of lentiviral vectors expressing EGFP under the control of EF1α or MeP promoter. RRE: Rev-Responsive Element, cHS4: chicken *β–globin* locus Hypersensitive Site 4, cPPT: central Poly Purine Tract, CTS: Central Terminal Sequence. B) Lenti-EF1α-EGFP vector was infected into the *ex vivo* brain slice from a wild-type mouse, and the EGFP expressing cells were detected by live imaging for up to 3 weeks after infection. C) Endogenous MeCP2 expression (green) in nuclei of cortical neurons (Tubulin III positive). D) Cortical neurons were infected *in vitro* with the lentiviral vectors expressing EGFP under the control of EF1α promoter (left) or MeP promoter (right). Percentage of EGFP positive cells (green line) were assessed by flow cytometry. E) Both EF1α (left) and MeP (right) promoter expressed EGFP in Tubulin III positive cortical neurons after infection of lentiviral vector. Scale bars represent 50 µm.

**Figure 10:**
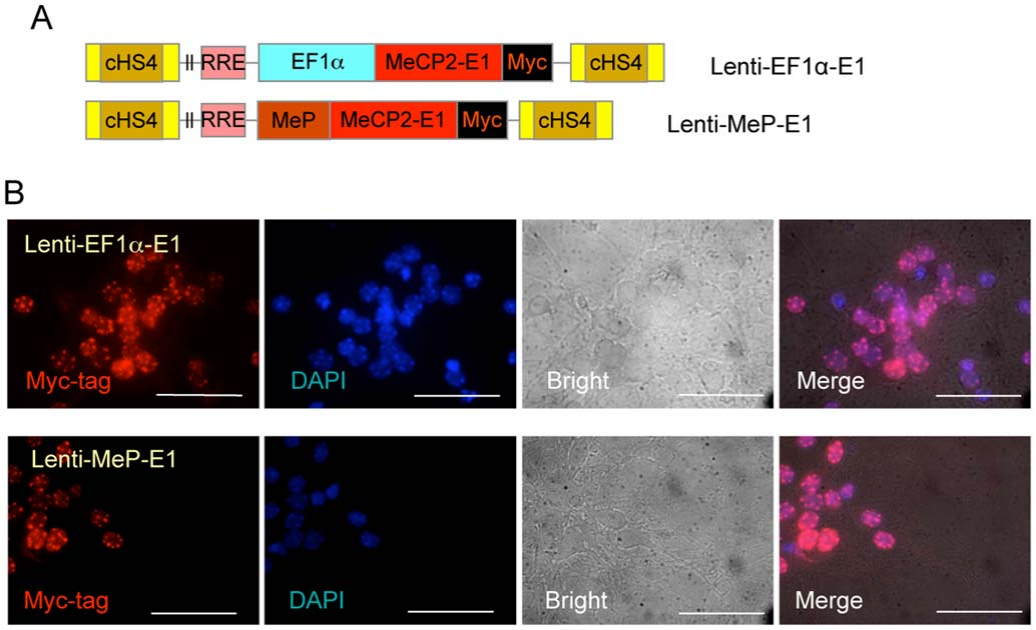
Efficient transduction of cortical neurons by lentiviral vectors expressing MeCP2 isoform. A) Schematic of lentiviral vectors expressing human MeCP2 isoform (E1) with Myc-tag under the control of EF1α or MeP promoter. B) Expression of Myc-tagged MeCP2-E1 colocalized with DAPI-rich regions in the nuclei of cortical neurons after infection with EF1α (top) and MeP (bottom) promoter lentiviral vectors. Scale bars represent 50 µm.

We next tested the ability of the lentiviral vectors to transduce differentiated neurons directly. In brain slice cultures, Lenti EF1α-EGFP (Figure 9A) infected morphologically differentiated cells with high efficiency and EGFP signals were maintained for 3 weeks (Figure 9B). Next, neurons were isolated from the brain cortex of E18 mouse embryos and cultured up to 72 hours prior to infection. These cells express endogenous MeCP2 as expected (Figure 9C). They were infected with concentrated lentiviral vectors and assayed for EGFP expression after 72 h. Flow cytometry revealed that >80% and >70% of cells infected with Lenti-EF1α-EGFP and Lenti-MeP-EGFP, respectively express EGFP (Figure 9D) as confirmed by IF (Figure 9E). Finally, Lenti-EF1α-E1 and MeP-E1 delivery (Figure 10A) also resulted in a high percentage of neurons expressing nuclear Myc-tag colocalized with DAPI (Figure 10B). Overall, our lentiviral vectors direct long-term *MECP2* isoform expression in undifferentiated NSC and/or their progeny neurons and glia, directly infect dissociated neurons with high efficiency, and therefore are well suited for future applications in RTT gene therapy.

## Discussion

### Long-term expression of EF1α-MECP2 vectors in adult and embryonic NSC

Gene therapy seeks to obtain stable and long-term expression of the engineered gene in transduced cells and their progeny. Stem cells are commonly used because of their ability to differentiate and generate a broad range of different cell types. However, one obstacle in stem cell applications for gene therapy is silencing of transduced genes. We generated SIN retroviral vectors using the HSC1 backbone to avoid all known silencer elements, and expressed EGFP and *MECP2* isoforms under the control of the strong ubiquitous EF1α promoter. We transduced NSC with high efficiency of about 80% for *MECP2* isoforms and showed long-term expression over a period of 10 passages without evidence for silencing of these advanced vector designs in NSC. Through *ex vivo* NSC injection, continuous expression of the transduced genes in the brain microenvironment of WT and *Mecp2^tm1.1Bird^*+/− female mice was monitored and observed for 3 weeks. Moreover, NSC from adult *Mecp2^tm1.1Bird^*+/− female mice maintained a stable frequency of expressing cells from the first to the 10^th^ sphere. Our data supports the finding that NSC from these mice have similar sphere forming capacity as WT adult NSC [32], and the infection efficiency of retroviral vectors are comparable. Overall, we show stable and highly efficient retroviral transduction of NSC.

### Restricted MeP-MECP2 expression in neurons and glia

For gene therapy of human disorders, regulated expression of the transferred gene is generally preferred. *MECP2* expression is under tight developmental regulation, which is required for proper postnatal brain development. In mice, *Mecp2* overexpression in post-mitotic neurons leads to profound motor dysfunction [29] and mild 2-fold overexpression is accompanied by progressive neurological disorders and premature death [36]. However, transgene expression in the mutant background rescues the phenotype [29]. It is therefore important to employ the endogenous *Mecp2* promoter in any RTT gene therapy attempt.

In our initial studies, we used the EF1α promoter and showed highly efficient NSC transduction with our retroviral vectors. Importantly, MeCP2 expression was directed to the correct subnuclear localization at heterochromatic regions of the nucleus. Overexpression of *MECP2* did not overtly interfere with the renewal capacity of NSC and these expressing cells were passaged up to the 10^th^ spheres with no difficulty. We then demonstrated endogenous MeCP2 expression in differentiated neurons and to a lower level in GFAP+ cells derived *in vitro* and *in vivo* as recently reported [19]. To mimic this endogenous expression pattern, we created novel *MECP2* isoform-specific vectors regulated by the endogenous MeP promoter that directed MeCP2 expression in neurons [30] and also glia [33]. Thus the MeP promoter vector recapitulates the endogenous expression pattern and is well suited for RTT gene therapy. To ensure that appropriate levels of MeCP2 are expressed from the viral vectors, it will be important to compare protein levels of endogenous and exogenous MeCP2 by performing additional experiments. These may require *MECP2* transgenes with a larger tag to distinguish the exogenous isoforms from endogenous MeCP2 by protein size.

### MeCP2-E1 expression in +/− NSC and function in neuronal maturation

As a model for gene therapy, we differentiated NSC from *Mecp2^tm1.1Bird^*+/− female mice at the 5^th^ sphere passage and observed endogenous MeCP2 expression in roughly 50% of the cells as expected. However, when +/− NSC were infected with the Retro-EF1α-E1 and Retro-MeP-E1 vectors and differentiated, between 95–100% of the cells were MeCP2-positive. These data show that retrovirus vectors expressing from the EF1α ubiquitous or MeP restricted promoters can convert virtually all +/− NSC into MeCP2+ cells.

To examine the function of the MeCP2-E1 isoform, Retro-EF1α-E1 infected +/− NSC were differentiated into neurons and morphological features of neuronal maturation determined. Significant increases in the length of primary dendrites, and increased numbers of secondary and tertiary dendrites were observed. These data are in agreement with the report that *Mecp2-*null neurons have fewer dendrites and impaired synaptic formation [37] and that *Mecp2*-null GFAP+ astrocytes stunt dendrites in cocultured neurons [19]. The *Mecp2-*E1 isoform is the dominant transcript detected in the mouse brain, and therefore its role in dendrite formation and maturation in neurons, or its supporting role in astrocytes, can now be examined *in vivo* using our isoform-specific retrovirus vectors.

### Lentiviral MECP2 delivery directly into neurons

In order to deliver MeCP2 isoforms directly into affected neurons, we employed a safety-enhanced SIN lentiviral vector that includes a dimer cHS4 core insulator element to prevent potential insertional activation events. These lentiviral vectors express EGFP and *MECP2* isoforms from the EF1α and MeP promoters in NSC, neurons and glia. As expected, MeCP2 was localized to heterochromatic regions of the nucleus. Importantly, the lentivirus vectors infected dissociated cortical neurons with high efficiency of 80% as well as brain slice cultures. Overall, our MeP lentiviral vectors restrict MeCP2 expression to the neuronal lineage, and prevent ectopic expression from occurring in NSC through their ability to directly transduce affected neurons and glia. One complication of direct lentivirus delivery is that X chromosome inactivation in female patients leads to half the cells expressing wild-type protein and half expressing mutant protein. Lentivirus transduction will express the human isoform in all infected cells. To address this issue, exogenous *MECP2* expression could be coupled to specific knockdown of the endogenous MeCP2 to preserve total expression levels in heterozygous patient cells [38].

### Routes for RTT gene therapy

We propose that *MECP2* gene transfer for RTT gene therapy can employ two routes. First, MeP retrovirus vectors can modify NSC that subsequently differentiate into cells that express MeCP2 in the endogenous pattern. This approach is dependent on having access to patient-specific NSC. To this end, recent advances deriving induced pluripotent stem (iPS) cells from human fibroblasts [39]–[41] now permit generation of patient-specific iPS cells [42]–[44]. In fact, we recently reported the generation of RTT-model mouse derived iPS cells and RTT-patient derived iPS cells [44]. Conceivably, these cells could be used to model RTT disease and as recipients for transduction with the MeP retrovirus or insulated lentivirus vectors. Differentiation of genetically corrected patient-specific iPS cells [45] might ultimately produce NSC grown as neurospheres that could be dissociated for transplantation to generate both neurons and their supportive glial cells. Second, the MeP lentivirus vectors can deliver the restricted *MECP2* transgene directly into neurons and glia. This approach could be applied *in vivo* by direct virus injection into the chosen regions of the brain depending on the severity of the symptoms of each patient [46], [47]. Alternatively, the MeP promoter constructs could be delivered more broadly across the Blood Brain Barrier through the use of Adeno-Associated Virus 9 derived vectors [48]. The feasibility of these approaches will need to be tested in mouse models *in vivo*.

In summary, we generated *MECP2* isoform-specific vectors and demonstrate their long-term expression in transduced embryonic and adult murine NSC. These viruses are not subject to stem cell-specific viral silencing due to the advanced vector design and maintain the expression of the transduced genes after neuronal differentiation. Our MeP vectors recapitulate endogenous MeCP2 expression in neurons and glia and rescue mosaic MeCP2 expression in *Mecp2^tm1.1Bird^*+/− neurons. We clearly demonstrate a functional role for MeCP2-E1 in regulating dendrite length and branching during morphological maturation of *Mecp2^tm1.1Bird^*+/− neurons *in vitro*. Our advanced lentiviral EF1α vectors efficiently transduce *MECP2* into cortical neurons and direct it to the proper nuclear compartments. These vectors are invaluable tools facilitating functional studies of MeCP2 isoforms and have important applications for RTT gene therapy.

## Materials and Methods

### Vector construction

pcDNA3.1A-*MECP2A*(E2*)-*myc and pcDNA3.1A-*MECP2B*(E1)-myc vectors containing human *MECP*2-isoform cDNAs with Myc-tag were kind gifts from Berge Minassian (Hospital for Sick Children). We subcloned *MECP2* isoforms into the HSC1-EF1α-EGFP retrovirus vector [49]. The DNA was transformed into SCS110 competent cells (Stratagene) to allow digestion with the dam-methylation sensitive enzyme *Cla*I, blunted with T4 DNA polymerase (Invitrogen) and digested with *Nco*I. The vector backbone was 5′ dephosphorylated using CIAP (Invitrogen) and blunt ligated to 1.5 kb *MeCP2*-E2 released from pcDNA3.1A-*MECP2A*(E2)-myc digested with *Pme*I and *Nco*I. Diagnostic digestion with *Nco*I and *Sac*I identified Retro-EF1α-E2 (same as HSC1-EF1α-*MECP2*E2-myc) was further confirmed with *Sma*I and *Hin*dIII. Retro-EF1α-E1 (same as HSC1-EF1α-*MECP2*E1-myc) was generated by PCR amplification of *MECP2*E1-myc from pcDNA3.1A-*MECP2B*(E1)-myc to introduce *Nco*I (5′) and *Cla*I (3′) sites to allow direct sub-cloning into the HSC1-EF1α vector [forward primer: 5′-GAGCTCGGATCCGGACCATGGC-3′ (*Nco*I site underlined); reverse primer 5′-CGATCGATAACTCAATGGTGATG-3′ (*Cla*I site underlined)]. The PCR product was cloned into TOPO 4.0 TA (Invitrogen) and confirmed by automated sequencing. It was digested by *Nco*I and *Cla*I, and cloned into Retro-EF1α-EGFP (same as HSC1-EF1α-EGFP) digested with *Nco*I and *Cla*I (to release the EGFP gene). Digestion with *Nco*I and *Sac*I identified the clone and confirmed by *Sma*I and *Hin*dIII digestion.

The MeP promoter was generated by PCR using genomic DNA from J1 ES. The forward primer had an *Xba*I site followed by the −678 to −656 of the MeP promoter: 5′-AGTCAGTCTAGATCTCTTATGGGCTTGGCACAC-3′. Reverse primer included an *Nco*I site positioned over the ATG start codon (E1 and E2 use the same ATG start codon) extending to +27: 5′-AGTCAGCCATGGTTTCCGGACGGGTTTTACC-3′. The PCR product contains an *Xba*I site followed by −678 bp to an *Nco*I site over the ATG start codon of the *MeP-Mecp2* region. The PCR product was cloned into pGEM-T-easy Vector (Promega). It was subsequently double digested using the *Eco*RI site of the pGEM-T-easy vector and the *Nco*I site introduced by PCR. The MeP promoter fragment was gel purified with Qiagen Gel Extraction Kit. This resulted in the introduction of an *Eco*RI, *Spe*I and *Xba*I site 5′ to the MeP promoter sequence. Next our Retro-EF1α-E1 and Retro-EF1α-E1 vectors were digested with *Nco*I and *Eco*RI and gel purified. The *Eco*RI-*Spe*I-*Xba*I-(−678)-Mep-(ATG)-*Nco*I fragment encoding the MeP promoter was ligated with the PL-cHS4 lentivirus vector backbone described previously [35]. The lenti-MeP-EGFP construct was obtained by ligating the 700 bp MeP fragment (Retro-MeP-EGFP; *Nco*I, *Bgl*II) and the 1.6 kb EGFP fragment (Lenti-EF1α-EGFP; *Eco*RI, *Nco*I) into the 5.2 kb Lentivirus backbone (Lenti-EF1α-EGFP; *Eco*RI, *Bam*HI). Lenti-EF1α-E1 and Lenti-EF1α-E2 constructs were obtained by ligating the 1.8 kb *MECP2*E1 and 1.8 kb *MeCP2*E2 fragments respectively (Retro-EF1α-E1 and Retro-EF1α-E2; *Nde*I treated with Klenow and subsequently *Xho*I) into the 7.1 kb lentivirus backbone (Lenti-EF1α-EGFP (*Nru*I, *Xho*I). Lenti-MeP-E1 and Lenti-MeP-E2 constructs were obtained by ligating the 700 bp MeP fragment (Retro-MeP-EGFP; *Nco*I, *Bgl*II) and the 2.4 kb *MeCP2E1* and 2.4 kb *MeCP2*E2 fragments respectively (Lenti-EF1α-E1 and Lenti-EF1α-E2; *Eco*RI, *Nco*I) into the 5.2 kb lentivirus backbone (Lenti-EF1α-EGFP; *Eco*RI, *Bam*HI**)**.

### NSC isolation, culture and differentiation

Mouse embryonic NSC were isolated from the forebrain of E14.5 embryos. Dissected tissues were collected by brief centrifugation, homogenized through a flame narrowed pasteur pipette, filtered through a 40 µm filter and plated at 10^5^ cells/cm^2^ in NSC media DMEM/F12 1∶1 (Wisent Inc.) in the presence of rhEGF (Sigma, 20 ng/ml), bFGF (Upstate, 20 ng/ml), Heparin (Sigma, 2 µg/ml) and hormone mix [50]. The neurospheres were dissociated to single cells every 7 days for sub cultures with Accutase treatment (Sigma; 1 ml, 2–5 min at 37°C), washed with basic media, filtered and cultured in 50∶50 fresh media and conditioned media (old NSC media). For differentiation, single cells were cultured in the presence of 10% FBS (Fetal Bovine Serum, Invitrogen) with no rhEGF or bFGF and grown on growth factor reduced Matrigel (BD) and the media was changed every other day. For adult mouse NSC, we dissected the Subventricular Zone (SVZ) of the forebrain into small pieces, collected by brief centrifugation, resuspended into 50 ml digestion mix [Hi/Low ACSF plus Trypsin (Sigma), 1.33 mg/ml; Hyaloronidase (Sigma), 0.67 mg/ml; Kynurenic acid (Sigma), 0.1–0.17 mg/ml] at 37°C (30 minutes). Tissue was collected by centrifugation and resuspended in 10 ml antitrypsin (Ovalbumin 0.7 mg/ml, Sigma), centrifuged and resuspended in 2 ml of full NSC media. The tissue was homogenized with a flame narrowed pasteur pipette about 40 times and plated at low density. Subculturing and differentiation was similar to the embryonic NSC.

### Retroviral production and NSC transduction

Retroviruses were prepared in Phoenix ecotropic packaging cells cultured in Dulbecco's modified Eagle's medium (DMEM, Invitrogen) with 10% FBS using Lipofectamine 2000 (Invitrogen) and 8 µg of retroviral DNA. The next day the media was replaced with NSC media and the virus was harvested 24 h later for NSC infection. Dissociated NSC were filtered, counted and were infected overnight with freshly made virus (1∶1 virus: media fresh media) in the presence of 6 µg/ml Polyberene (Sigma). The virus was removed the next day and the cells were plated in fresh media.

### Lentiviral production

Lentivirus vectors were produced in 293-T cells cultured in DMEM with 10% FBS. For lentiviral production, 293-T cells were plated at a density of 8×10^6^ in T-75 flasks. The following day, the cells were transfected using Lipofectamine 2000 (Invitrogen) with 10 µg HPV275 (gag/pol expression plasmid), 10 µg P633 (rev expression plasmid), 10 µg HPV17 (tat expression plasmid), 5 µg pHCMV-VSV-G (VSV-G expression plasmid) and 15 µg of PL-cHS4 based *MECP2* lentivirus vectors. The lentiviruses were collected in 20 ml media after 481h, filtered through 0.45 µm pore filters and concentrated by ultracentrifugation at 4°C, 2 h, 30,000 rpm with T-865 rotor (Sorvall). The pellet was resuspended to final volume of 80 µl with Hanks' balanced salt solution (HBSS, Invitrogen) overnight at 4°C. Next day, 10^5^ target cells were infected with lentiviruses at different doses (4, 10 or 40 µl) in the presence of 8 µg/ml polybrene. After 24 h, the media was replaced with fresh medium. Virus titers were estimated by flow cytometry using the formula: N×M / 100 V; N is the number of target cells used for infection, M is % expressing cells, and V is the volume of concentrated virus used (ml).

### Brain slice culture

The brain of WT or *Mecp2^ tm1.1Bird^+/−* female mice (The Jackson Laboratory) were dissected in HBSS and sliced with a tissue chopper (McIlwain, Campdan Instruments, Lafayette, IN) into coronal sections of about 300 µm [51] prior to infection with lentiviruses or injection with transduced NSC. Intact slices were selected and transferred onto a millicell sterilized 0.4 µm culture plate insert (Millipore), then placed into a six-well culture plate containing 700 µl of media, composed of basal medium Eagle's (Invitrogen), with 25% vol/vol Hank's Balanced salt solution (Invitrogen), 25% vol/vol heat inactivated horse serum (Invitrogen), 0.2% wt/vol glucose (culture grade, Sigma) and 0.65% wt/vol sodium bicarbonate (culture grade, Sigma). Approximately 5 brain sections were placed per insert, and media was changed every 3 days. Injections were done under a dissection microscope using a Hamilton syringe and pulled capillary tubes. For NSC injections, 1 µl of dissociated neurospheres with a concentration of 100,000 cells/µl was injected into the subventricular zone. For lentiviral infection, 20 µl of concentrated virus was added to the media and 20 µl of the virus was added to the top of the brain slice as a drop in the presence of 1 µg/ml polybrene overnight, with media changed the next day.

### Neuronal dissociation and infection

Postmitotic cortical neurons were isolated from E18 mouse embryos [52] and cultured on cover-slips in Neurobasal media (Invitrogen) supplemented with B27 (Invitrogen). After 3 days the cells were infected with 1 µl of concentrated lentiviruses (with 0.6 µg/ml polybrene). The virus was removed after 5–7 h and the cells were incubated with fresh media. After 48 h the cells were fixed and processed for IF.

### Neuronal tracing

We used ImageJ software (http://rsb.info.nih.gov/ij/) with NeuronJ plugin software (http://www.imagescience.org/meijering/software/neuronj/) for neuronal tracing and measurements. We quantified the axonal and dendrite length of 6 neurons in control and 11 neurons in infected cells, due to the small number of isolated cells that we could trace. *P* values for all the quantifications were measured and are presented as * *P*<0.05 and ** *P*<0.005.

### Immunostaining

Dissociated cells were plated on cover-slips coated with growth factor reduced Matrigel for undifferentiated/differentiated NSC or Poly-L-lysine (Sigma) for cortical neurons. Cells were washed twice with Phosphate Buffered Saline (PBS, Invitrogen) and fixed in 100% methanol at −20°C for 30 min. Cover slips were air dried and kept at −20°C until stained. For staining they were rehydrated in PBS (5 minutes), blocked 1 h at room temperature (RT) with 10% normal goat serum (NGS) in PBS and incubated with the primary antibody in 10% NGS, 1 h RT. They were then washed three times with PBS (5 minutes), incubated with the secondary antibody in 10% NGS for 1 h at RT, washed three times with PBS and mounted in antifade and DAPI (0.5 µg/ml).

### Antibodies

The following antibodies were used: anti-C-Myc, (Rabbit, SC789 Santa Cruz, IF 1∶200; WB 1∶500); anti-C-Myc (Mouse, A21280 Molecular Probes, IF 1∶200; WB 1∶500); anti-GFAP (Mouse, A21282 Molecular Probes, IF 1∶200); anti-MeCP2 (Rabbit, 07-013 Upstate, IF 1∶200); anti-Tubulin βIII (Mouse, MAB1637 Chemicon, IF 1∶200); anti-GFP Alexafluor-488 (A21311 Molecular Probes, IF 1∶400); anti-Actin (Mouse, Sigma, AC15).

### Total cell extracts and Western Blot (WB)

Total cell extracts were prepared as described previously [53] and WB was done with 20 µg of total cell extracts as described elsewhere [53].

#### Flow cytometry

Flow cytometry was performed using a FACScan (Becton–Dickinson) in the Hospital for Sick Children Flow Cytometry Facility and analyzed with FlowJo software.

## Supporting Information

Figure S1 NSC migration into the brain microenvironment in *ex vivo* culture. EGFP expressing NSCs (infected with Retro-EF1α -EGFP) were injected into brain slices of wild-type mouse, and EGFP expressing cells were detected by live imaging at the indicated days after injection.
(1.46 MB EPS)

Figure S2 Generation and maintenance of NSCs from *Mecp2^tm1.1Bird^*+/− female mice. A) *Mecp2^tm1.1Bird^*+/− female mouse displayed RTT symptoms such as hind limb clasping and small brain (inset, scale bar 5 mm). B) Neurospheres were generated from *Mecp2^tm1.1Bird^*+/− female brain. NSCs were maintained up to 21 passages for non-infected control (left), and 20 passages for Retro-EF1α-E1 infected control (right).
(1.69 MB EPS)

Figure S3 MeCP2 delivery into NSC by lentiviral vectors. A) Schematic of lentiviral vectors expressing human MeCP2 isoforms (E1 or E2) with Myc-tag under the control of EF1α or MeP promoter. B) Dissociated NSCs were infected with indicated lentiviral vector expressing MeCP2 isoforms. Immunofluoresence staining shows colocalization of Myc-tag and MeCP2 signals in DAPI-rich region from the EF1α promoter. No transgene expression (Myc-tag) was detected from the MeP promoter in NSCs, as with endogenous mouse MeCP2. Scale bars represent 50 µm.
(4.42 MB EPS)

Figure S4 MeCP2 isoforms were detected by WB in NSCs after lentiviral vector infection. NSCs were infected with indicated lentiviral vectors and whole-cell lysates were extracted from 1st sphere (1st Sph.) and 5th sphere (5th Sph.). Expression of MeCP2 isoforms was detected by WB using anti Myc-tag antibody from the EF1α promoter, but not from the MeP promoter.
(1.02 MB EPS)

Figure S5 MeP promoter is active in neurons and glia after differentiation of NSCs. Infected NSCs with indicated lentiviral vector were differentiated for 14 days. Immunofluorescense images show nuclear localization of exogenous MeCP2 protein (Myc-tagged) in Tubulin III positive neurons and GFAP positive glia. Scale bars represent 50 µm.
(2.21 MB EPS)
